# Machine Learning Applications in Sarcopenia Detection and Management: A Comprehensive Survey

**DOI:** 10.3390/healthcare11182483

**Published:** 2023-09-07

**Authors:** Dilmurod Turimov Mustapoevich, Wooseong Kim

**Affiliations:** Department of Computer Engineering, Gachon University, Sujeong-gu, Seongnam-si 461-701, Gyeonggi-do, Republic of Korea; dilmurod@gachon.ac.kr

**Keywords:** sarcopenia, AWGSOP, EWGSOP, physical performance, ML algorithms

## Abstract

This extensive review examines sarcopenia, a condition characterized by a loss of muscle mass, stamina, and physical performance, with a particular emphasis on its detection and management using contemporary technologies. It highlights the lack of global agreement or standardization regarding the definition of sarcopenia and the various techniques used to measure muscle mass, stamina, and physical performance. The distinctive criteria employed by the European Working Group on Sarcopenia in Older People (EWGSOP) and the Asian Working Group for Sarcopenia (AWGSOP) for diagnosing sarcopenia are examined, emphasizing potential obstacles in comparing research results across studies. The paper delves into the use of machine learning techniques in sarcopenia detection and diagnosis, noting challenges such as data accessibility, data imbalance, and feature selection. It suggests that wearable devices, like activity trackers and smartwatches, could offer valuable insights into sarcopenia progression and aid individuals in monitoring and managing their condition. Additionally, the paper investigates the potential of blockchain technology and edge computing in healthcare data storage, discussing models and systems that leverage these technologies to secure patient data privacy and enhance personal health information management. However, it acknowledges the limitations of these models and systems, including inefficiencies in handling large volumes of medical data and the lack of dynamic selection capability. In conclusion, the paper provides a comprehensive summary of current sarcopenia research, emphasizing the potential of modern technologies in enhancing the detection and management of the condition while also highlighting the need for further research to address challenges in standardization, data management, and effective technology use.

## 1. Introduction

Sarcopenia, a condition characterized by the loss of muscle mass, strength, and physical performance, is a significant health concern, particularly in older populations. As the global population ages, the prevalence of sarcopenia is expected to rise, leading to increased healthcare costs and degradation in the quality of life (QoL) for affected individuals. Accordingly, the prediction and management of sarcopenia disease become critical for public health all over the world. In this survey paper, we first explore sarcopenia in depth and propose a healthcare platform with applicable technologies for its detection and management.

There is no unified criterion globally for sarcopenia diagnosis. This lack of consensus in sarcopenia’s definition and diagnostic criteria can be a hurdle to developing effective interventions and treatments. Instead, several methodologies exist, such as muscle mass or strength measurement and physical performance tests. For this, the European Working Group on Sarcopenia in Older People (EWGSOP) and the Asian Working Group for Sarcopenia (AWGSOP) each define criteria for diagnosing sarcopenia.

The measurement of muscle mass is crucial for the diagnosis of sarcopenia. Instruments including Dual-energy X-ray absorptiometry (DEXA), computed tomography (CT), and magnetic resonance imaging (MRI) are popularly used to measure muscle mass. However, the diagnostic outcomes can vary by each instrument with disparate sensitivity and intrinsic specificity, and the threshold values for diagnosing sarcopenia via these imaging modalities are absent of unique standards. Furthermore, diagnostic equipment may not be readily available in primary care settings, which can obstruct the early detection of individuals at risk of developing sarcopenia and following therapeutic management.

The early detection of sarcopenia is crucial as it allows for the implementation of interventions that can slow the progression of the condition, improve the quality of life for affected individuals, and reduce healthcare costs.

Recent literature demonstrates considerable interest in evaluating determinants of sarcopenia and devising methodologies to precisely quantify these determinants [1]. Key determinants include the degree of physical exertion, prolonged sedentary behavior, cardiopulmonary endurance, and muscular fortitude. However, accurately measuring these determinants presents challenges. Self-reported data are commonly utilized to assess physical exertion but may be subjective and biased. Moreover, while fitness examinations provide objective data, they may not be feasible across all settings. Therefore, developing valid and reliable metrics to evaluate physical activity and fitness levels is critical for identifying and intervening in sarcopenia.

Machine learning techniques show promise for predicting sarcopenia, as demonstrated through Support Vector Machines (SVM), Random Forests (RF), and Gradient Boosting Machines (GBM) models. These algorithms can analyze large datasets and detect patterns predictive of sarcopenia onset and progression. However, applying machine learning for sarcopenia detection faces obstacles like data accessibility, imbalance, and feature selection. High-quality, representative training and testing data are needed but scarce [2]. Imbalanced class distributions can skew predictions. Identifying predictive features while avoiding overfitting remains challenging. Thus, effective data collection, balancing, and feature selection strategies are vital when utilizing machine learning to detect sarcopenia.

Wearable devices offer the potential for tracking sarcopenia by continuously monitoring physical activity and health indicators. The resulting data could reveal muscle mass, strength, and function changes to identify and manage sarcopenia. However, wearables also present data accuracy, interpretation, and user adherence limitations that warrant consideration before deployment.

Various models employ blockchain technology and edge computing to enhance privacy protection and data sharing for personal health records, promoting security, confidentiality, and interoperability. Nonetheless, current frameworks have shortcomings in handling large medical datasets and dynamically selecting resources that require improvement.

Despite extensive research, major gaps remain in sarcopenia prevention, diagnosis, and management. There is no consensus on its operational definition or cut-off values for key parameters like muscle mass and strength. Multiple assessment techniques like imaging, physical performance tests, and questionnaire tools are employed, but their integration into diagnostic and monitoring frameworks needs optimization. Rapid advancements in technologies like wearable sensors, machine learning, and mobile health apps offer tremendous potential to enhance sarcopenia detection and care but also present analysis, validation, and implementation challenges.

This comprehensive survey aims to synthesize insights from recent studies to elucidate the current state of sarcopenia research. It explores promising technical approaches for assessment and diagnosis but also critiques their limitations to shed light on areas needing further investigation. The survey delves into sophisticated machine learning techniques but grounds their utility through a careful examination of challenges related to data accessibility, feature selection, and model optimization. It highlights innovative sensor-based tools while weighing factors influencing their accuracy, adoption, and integration into platforms or interventions.

In consolidating evidence regarding sarcopenia, this survey identifies remaining knowledge gaps, technology limitations, and standardization needs. It provides a launch point to motivate key next steps in strengthening definition consensus, assembling predictive datasets, advancing analytics, and translating technologies into patient care. Most importantly, it aims to guide future sarcopenia research toward targeted efforts that move from conceptual frameworks toward pragmatic clinical solutions. This paper seeks to inspire tangible progress in screening, diagnosis, and management to reduce the encroaching public health burden of this complex geriatric condition.

This survey synthesizes current evidence on the topic of interest. The literature search used Google Scholar, Web of Science, and ScienceDirect databases. Peer-reviewed papers published in English within the last three years were prioritized for inclusion, see Table 1. Both original research studies and review articles were eligible for inclusion if they provided relevant insights on the topic. Key data and findings were extracted from the selected papers using a standardized form to collate and compare results. Information was synthesized by grouping papers into themes and chronologically analyzing research trends. The main limitation of this approach is that the literature search may not be as comprehensive without predefined search strategies across multiple databases. However, this survey still provides a qualitative synthesis of existing evidence on the topic.

Compared to the survey paper by Pawan and Praveen [3], the current review provides a more comprehensive overview of sarcopenia detection and management leveraging modern technologies. While [3] focused predominantly on diagnostic criteria and physical performance assessments, the current paper delves deeper into technological innovations like machine learning, wearable devices, blockchain, and edge computing for sarcopenia. It highlights advancements in applying techniques like SVM, random forests, and gradient boosting for prediction. The discussion on wearables, mobile apps, insoles, and IMUs for continuous monitoring and assessment is more detailed.

Moreover, the current review uniquely covers emerging topics like blockchain for secure health data exchange and edge computing for localized processing absent in [3]. However, the machine learning section in [3] explores supervised learning algorithms more extensively. Overall, the current paper provides better insights into technology-enabled sarcopenia management with a broader scope.

The structure of this paper is as follows. Section 2 delves into the intricacies of physical performance with respect to sarcopenia, while Section 3 explores the criteria for diagnosing this condition. Digital health technologies and IoT devices specifically crafted for sarcopenia are the subject of Section 4. A thorough examination of the healthcare platform underpinning these IoT frameworks is given in Section 5, while Section 6 offers an overview of the machine learning algorithms applicable in the context of sarcopenia. Section 7 uncovers the challenges associated with applying these technologies in the management of sarcopenia. Finally, the paper draws to a close with Section 8, summarizing the key points and offering concluding thoughts.

## 2. Physical Performance Test for Sarcopenia

Sarcopenia, a progressive and generalized skeletal muscle disorder, leads to adverse outcomes such as falls, disability, cognitive decline, and mortality and a significant threat to the maintenance of independence [4], which can be estimated by a decrease in muscle mass and strength, and physical performance.

### 2.1. Grip Strength

Hand grip strength (HGS), as a surrogate for muscle strength, is often assessed to indicate muscle functions in the context of sarcopenia [5]. HGS is useful for identifying individuals with low muscle strength as an initial step in diagnosing sarcopenia according to universal cut-off points for grip strength recommended by the European Working Group on Sarcopenia in Older People (EWGSOP2) [4].

Conventionally, HGS has been measured by a dynamometer and measurement protocols. Recently, innovative approaches have been proposed indirectly to measure grip strength. Jeong et al. [6] adopted joint angles of fingers from finger tracking to measure hand grip strength, which could potentially be developed into a mobile application, showing a low error rate of less than 15% in measuring grip strength. Also, Barrios et al. [7] introduced a simple mobile application measuring finger tapping speed, which is related to grip strength.

However, grip strength measured by those methods can vary and cause a wide range of cut-off points in the screening for sarcopenia [8,9]. Furthermore, grip strength can be different across countries, which is expected to impact the prevalence of sarcopenia. Accordingly, the choice of HGS criterion (average vs. maximum) significantly impacted the prevalence of low HGS and sarcopenia, and their predictive validity for physical performance [10]. Similarly, De et al. [11] argued that HGS as an independent predictor of sarcopenia should only be used as a screening tool to stratify the need for confirmatory CT-based assessment of sarcopenia.

For understanding differences in grip strength by country, age, and sex, standardization of HGS measurements and criteria are needed for sarcopenia diagnosis. Therefore, several studies have attempted to standardize the measurement of grip strength and determine cut-off points. For instance, the “Southampton protocol” proposed by Roberts et al. [12] and a systematic review by Schaap et al. [13] were introduced to standardize the measurement of grip strength [5]. Despite these efforts, various factors such as racial diversity, body size, lifestyle differences, and socioeconomic status can still influence grip strength and thus the cut-off points for diagnosing sarcopenia [9].

Alternatively, other bio evidence can be used to indirectly estimate lower grip strength and muscle power. For instance, an increment in plasma asymmetric dimethylarginine (ADMA) level can be measured and is significantly associated with low grip strength and sarcopenia [14]. In [15], serum proteomics analysis was used to identify novel biomarkers for the diagnosis of sarcopenia, which found a total of 114 deferentially expressed proteins (DEPs) between the patients and healthy older adults, including 48 upregulated proteins and 66 downregulated proteins, and identified cholesterol ester transfer protein and Apolipoprotein A2 as potential biomarkers that are related to low muscle power.

### 2.2. Short Physical Performance Battery (SPPB)

The Short Physical Performance Battery (SPPB) is a widely utilized and validated tool for evaluating physical performance in older adults and has been extensively applied in the diagnosis and assessment of sarcopenia [16,17]. Multiple studies have utilized the SPPB to elucidate the relationships between sarcopenia, physical performance, and health outcomes, providing valuable insights into the significance of this condition.

Population-specific investigations of the SPPB in Asian communities have established normative values that can potentially improve the precision of sarcopenia diagnoses. A meticulous assessment of SPPB parameters—balance, repeated chair sit-to-stand, and gait speed—resulted in average scores of 11.6 for men and 11.5 for women, which decreased with advancing age [16]. Other studies have validated the SPPB as an objective measure of muscle strength and physical performance for diagnosing sarcopenia, with a cut-off point of 8 having high sensitivity but lower specificity for severe sarcopenia [17].

The association between SPPB, sarcopenia, and falls resulting in fractures appears to have gender-specific distinctions, with low handgrip strength and SPPB scores influencing falls in males, while only low muscle mass influenced falls in females [18]. Sarcopenia and sarcopenic obesity also have differential effects in cardiovascular disease patients, implying distinct implications for cardiovascular health [19].

Higher thyroid hormone levels within normal ranges have been associated with improved muscle mass and SPPB performance, suggesting a potential role in preserving muscle function [20]. However, other studies indicate a complex relationship between adiposity, obesity, and sarcopenia parameters like handgrip strength and SPPB, with obesity potentially conferring some protective effects against sarcopenia in women [21].

The SARC-F questionnaire is another sarcopenia screening tool that correlates with SPPB scores and outcomes but may be limited by a ceiling effect [22]. The SPPB is an extensively utilized and validated assessment tool that has provided critical insights into sarcopenia and its relationships with physical performance and health outcomes. However, it has some limitations, indicating that a combination of methodologies may be optimal for sarcopenia assessment.

### 2.3. Gait Speed

Multiple studies have demonstrated that reduced gait speed is associated with sarcopenia and correlated with disease severity and functional impairment in older adults. Gait speed has emerged as a key indicator and potential screening tool for sarcopenia-related disability. This subsection synthesizes evidence from recent research on gait alterations indicative of sarcopenia, the relationship between gait velocity and sarcopenia severity, and the utility of instrumented gait analysis for assessing and monitoring age-related mobility decline.

In a large cohort study of nearly 20,000 older Colombian adults, Perez et al. [23] found that slower gait speed mediated the relationship between sarcopenia and dependence on activities of daily living (ADLs). After adjusting for confounders, the researchers concluded that maintaining gait velocity may help mitigate sarcopenia’s negative impact on functional status. Similarly, Liao et al. [24] reported delayed post-operative gait recovery in sarcopenic, obese, and sarcopenic obese patients following total knee replacement.

Several studies reveal specific quantitative gait changes associated with sarcopenia. Compared to healthy controls, sarcopenic individuals exhibit reduced step length, shortened stride length, slower walking velocity, shortened single limb support phase, and prolonged double support phase [25,26,27]. While cadence is often preserved, sarcopenic gait shows a shortened gait line, indicating reduced muscle mass [25]. Sarcopenic women had a significantly slower pace and shorter steps versus normal controls in one study [27].

Beyond simple speed, the variability and dual-task cost of gait may provide further insights into sarcopenia severity. Kang et al. [28] found sarcopenic elderly men walked significantly slower daily than non-sarcopenic peers. Dual-task assessments demonstrate greater slowing in individuals with sarcopenia versus single-task walking [29]. Greater dual-task costs are associated with increased fall risks and mobility impairment.

Wearable sensors enable continuous monitoring of gait parameters in real-world settings. Kang et al. [28] showed daily gait speed derived from wearables accurately reflected sarcopenia diagnosis. Machine learning models using combinations of gait features derived from wearables may enable sarcopenia detection and monitoring [29]. Compared to infrequent clinical gait tests, continuous monitoring could facilitate early identification of mobility decline.

While gait speed shows promise as an indicator of sarcopenia severity, optimal thresholds for screening remain unclear. Most diagnostic criteria define sarcopenia as gait speed below 0.8 m/s [30], but this cut-off may lack sensitivity. Machine learning applied to wearable gait data could potentially derive optimal speed thresholds and improve detection. Additional research should validate gait parameters and thresholds that best correlate with imaging-confirmed sarcopenia.

Though gait speed is consistently associated with sarcopenia, the underlying mechanisms remain incompletely understood. Reduced velocity may stem directly from the loss of muscle mass and strength. However, neurological and cognitive impairment also correlate with slowed gait in sarcopenic patients [31]. Disentangling the multiple interacting factors contributing to gait changes represents an area for further study.

Extensive evidence demonstrates reduced gait velocity and altered gait patterns across multiple clinical contexts in sarcopenic patients. Slower speed correlates with disease severity, risk of falls and fractures, and functional impairment. Instrumented gait analysis, especially using wearable sensors, provides a promising approach to screening for sarcopenia and monitoring age-related mobility decline. Future research should further validate optimal gait parameters and diagnostic thresholds. Preserving mobility through exercise interventions may help mitigate sarcopenia-related disability.

### 2.4. Timed Up and Go (TUG) Test

The Timed Up and Go (TUG) test is a commonly utilized assessment for evaluating physical functioning, sarcopenia, and frailty status in older adults and diverse clinical conditions [32,33]. This simple test measures the time required for an individual to rise from a chair, walk 3 m, turn around, walk back, and sit down.

Comparative studies have elucidated the relationships between TUG performance, gait speed, and sarcopenia indicators. TUG completion time demonstrates a nonlinear association with gait velocity and may be most useful for assessing individuals at the lower end of physical function, whereas gait speed assessments can evaluate performance across a wider spectrum [32]. For community-dwelling older adults, slower gait and poorer TUG performance are both associated with sarcopenic obesity [32].

In chronic obstructive pulmonary disease patients, poorer TUG test results are significantly associated with an increased likelihood of sarcopenia [33]. This highlights the test’s utility for identifying sarcopenic individuals. Similarly, longer TUG completion times are linked to higher sarcopenia risk in the general older population [34].

However, other studies indicate that TUG performance may not correlate with all sarcopenia indicators. Probable sarcopenia based on muscle mass and strength criteria was associated with slower gait but not impaired TUG performance [35]. Low muscle power, measured by chair rise testing, had stronger associations with functionality than sarcopenia classification itself [35].

Overall, the TUG test is a simple and clinically useful assessment tool that provides insights into sarcopenia likelihood and associated impairments in physical functioning. However, its relationships with specific sarcopenia parameters are complex. Recent research has focused on enhancing TUG assessment through quantitative movement analysis using inertial sensors, enabling more objective and detailed performance characterization [36]. Such technological augmentation of TUG testing shows promise for improving sarcopenia screening and monitoring. Nevertheless, the TUG test alone has limitations in comprehensively evaluating this multifaceted condition. Using TUG alongside other validated sarcopenia assessments can provide the most accurate insights.

### 2.5. Five Times Sit-to-Stand Test

The early detection and diagnosis of sarcopenia through validated assessment tools are critical for implementing timely interventions to curb the progressive loss of muscle mass and function in the geriatric population [37,38]. In this context, the Five Times Sit-to-Stand Test (5TSTS) has emerged as a clinically useful and valid instrument for evaluating functional trajectories in older adults. A key advantage of the 5TSTS is its capacity to detect sarcopenia in individuals who may be missed by conventional handgrip dynamometry, highlighting the need for a more comprehensive battery of tests [37].

As a simple, quick, and easy-to-administer test with minimal space, equipment, and time requirements, the 5TSTS provides valuable functional performance data to track sarcopenia progression [38]. Beyond sarcopenia, 5TSTS performance can identify fall risk, with times exceeding 13.5 s suggesting poorer lower limb muscle quality and fall propensity [39]. Intriguingly, sarcopenia prevalence is substantially higher when defined using the 5TSTS rather than handgrip strength, confirming its superiority for detecting age-related strength decline in the lower limbs [40].

The 5TSTS encapsulates multiple facets of lower body function, including strength, power, and dynamic balance. Test parameters such as ground reaction force, rate of force development, and chair rise time provide insight into strength and locomotor capacity [41]. As a measure of power, the 5TSTS is proposed as a simple but valid screening tool for age-related muscle weakness [42]. Superior diagnostic accuracy stems from the high power output needed to complete multiple rapid stands. Shorter 5TSTS times indicate greater muscle strength and power.

In summary, extensive evidence endorses the 5TSTS as a clinically useful assessment tool for sarcopenia screening and monitoring. Key advantages, including simplicity, minimal equipment needs, and detection of lower limb weakness, cement its role within a comprehensive geriatric assessment battery. Continued research is needed to refine diagnostic cut-off points and further validate their utility across diverse populations.

### 2.6. Physical Performance Test: Challenges and Future Work

Grip strength is a key component of sarcopenia diagnosis and an important biomarker in older adults, predicting outcomes such as mortality [43]. However, grip strength measurement lacks standardization across studies, including standardized testing protocols and weakness cut-off points [43,44]. Similarly, gait speed strongly predicts adverse health outcomes but also suffers from variability in cut-off points for sarcopenia diagnosis [23,27]. Although gait speed shows promise in assessing sarcopenia impact, with one study finding it may positively influence sarcopenia-related disability after adjusting for confounders [23], additional research is needed to determine optimal cut-off points.

The SPPB, commonly used to assess physical performance, also has limitations like the lack of universal cut-off points and classification accuracy for diagnosing sarcopenia [17,45]. However, the SPPB exhibits potential for sarcopenia screening when other assessments are unavailable [17]. The TUG test is another physical performance measure with promise for assessing sarcopenia [46,47] but requires the establishment of standardized cut-off points and further evaluation of feasibility in varied clinical settings before wider adoption.

Overall, current tools for sarcopenia assessment require additional research to determine standardized protocols and cut-off points tailored to different populations. Focusing on muscle function rather than mass alone may provide a greater prediction of outcomes. Emerging techniques like blood flow restriction training show promise in counteracting sarcopenia but require more study. Early screening in healthy young adults could help mitigate future sarcopenia onset. In conclusion, current sarcopenia assessment tools have limitations but hold promise with further refinement and research on optimal implementation.

## 3. Cut-Offs for Diagnosing Sarcopenia

The Asian Working Group for Sarcopenia (AWGS) and the European Working Group for Sarcopenia (EWGS) are devoted to the diligent scientific scrutiny and elucidation of sarcopenia. Characterized as the age-associated systemic decline of skeletal muscle mass and strength, sarcopenia signifies a health condition that entails numerous detrimental outcomes. The primary aim of these working groups is to formulate, refine, and disseminate strategies pertaining to the prevention, diagnosis, and clinical handling of sarcopenia.

However, it is imperative to acknowledge the multifaceted nature of sarcopenia. Managing and intervening in these conditions are swayed by a wide array of factors. Factors such as age, gender, race, ethnicity, and even geographical location play substantial roles in the inception and intensification of sarcopenia, as affirmed by multiple studies. Given such complexity, it is abundantly clear that sustained, comprehensive research is vital for attaining an all-encompassing understanding of the condition. This acquired knowledge is then indispensable in formulating efficacious, evidence-supported strategies for the management and intervention of sarcopenia.

### 3.1. Cut-Offs of Asian Working Group of Sarcopenia

The complexity and nuances of diagnosing and treating sarcopenia, characterized by an age-related loss of muscle mass and declining physical performance, have prompted the formulation of various operational definitions and guidelines. Among the key contributors to this discourse is the Asian Working Group for Sarcopenia (AWGS). The seminal work of Chen et al. [48] provides an insightful overview of AWGS’s updated approach, setting the group apart from its counterparts in a number of significant ways.

AWGS has been instrumental in introducing a series of influential updates in the sarcopenia landscape. These changes encompass a revised definition of sarcopenia, novel diagnostic criteria, and new guidelines for treating and managing the condition. Of particular importance is the recognition of low muscle mass as a fundamental indicator of sarcopenia, a shift that has significant implications for diagnosis. Moreover, the inclusion of gait speed as a functional parameter marks a pivotal step in the operationalization of the condition, taking into account the patient’s physical functionality in addition to their muscular characteristics. Further, the endorsement of resistance training as a primary treatment option underscores the importance of strength conditioning in managing and reversing the condition’s progression.

It is also noteworthy how AWGS’s perspective on diagnosing and treating sarcopenia distinguishes itself from other organizations. For example, the AWGS 2014 consensus characterized sarcopenia with explicit criteria, including an age-related loss of muscle mass coupled with low muscle strength and/or diminished physical performance. The AWGS also provides specific cut-offs for each diagnostic component, contrasting with other organizations adopting different definitions and diagnostic criteria.

A crucial point of distinction lies in AWGS’s emphasis on ethnic differences in muscle mass and function when diagnosing sarcopenia in Asian populations. This is an acknowledgment of the variability in physiological characteristics across different ethnic groups, a factor that plays a key role in the manifestation and progression of sarcopenia.

In summary, AWGS’s approach, as highlighted by Chen et al. [48], exhibits a number of unique aspects, including an updated definition of sarcopenia, the inclusion of functional parameters, and consideration of ethnic differences. These contributions significantly enrich the ongoing discourse on the effective diagnosis and treatment of sarcopenia.

AWGS offers a range of practical recommendations for healthcare professionals working with sarcopenic patients. These include the following:

1. Screening older adults for sarcopenia using a combination of muscle mass, muscle strength, and physical performance measures.
2. Implementing lifestyle interventions and providing related health education for primary healthcare and preventive service users with potential sarcopenia.
3. Referring patients to a hospital for a confirmatory diagnosis if sarcopenia is suspected.
4. Investigating potential underlying causes of sarcopenia, particularly reversible ones, in hospital and research settings.
5. Developing appropriate personalized intervention programs for older adults with sarcopenia, including resistance training, nutritional support, and pharmacological interventions if necessary.

These guidelines aim to assist healthcare professionals in effectively diagnosing and managing sarcopenia in older adults. Cut-offs, as per the Asian Working Group for Sarcopenia, include the following, see Table 2:

These cut-offs can be employed to diagnose sarcopenia based on low muscle mass, low muscle strength, and/or low physical performance criteria, as specified by the AWGS 2019 consensus update on sarcopenia diagnosis and treatment.

### 3.2. Cut-Offs of European Working Group of Sarcopenia

A key aspect in the geriatric healthcare domain involves the evaluation of potential predictors of mortality risk, particularly in the context of sarcopenia, a condition associated with the aging process. Recent studies have focused on identifying the most accurate predictors, and in doing so, they have called into question the effectiveness of existing diagnostic criteria. The research conducted by Spexoto et al. [49] serves as a prime example of this emerging narrative.

The European Working Group on Sarcopenia in Older People (EWGSOP) has developed two diagnostic frameworks for sarcopenia, namely EWGSOP1 and EWGSOP2. Notable differences between the two criteria lie in the cut-off values for muscle mass, the inclusion of gait speed as a diagnostic parameter, and the severity classification. Specifically, the EWGSOP1 criteria define Low Muscle Mass (LMM) and Low Muscle Strength (LMS) with higher cut-off values and Low Physical Performance as a gait speed < 0.8 m/s [49]. On the other hand, the EWGSOP2 employs more stringent cut-off values for LMM and LMS but maintains the same threshold for Low Physical Performance [49].

In their study, Spexoto et al. [49] undertook an expansive analysis of 6182 individuals aged 60 years and above within the English Longitudinal Study of Ageing. The outcomes of this study indeed add a new dimension to our understanding of mortality predictors.

Contrary to the LMS definitions predominant in the literature, the study identified cut-off values of <36 kg for men and <23 kg for women, in conjunction with a Low Gait Speed (LGS) ≤ 0.8 m/s, to demonstrate the highest accuracy for predicting mortality. This discovery brings forth an intriguing possibility that existing diagnostic criteria might not optimally represent mortality risk in older adults, see Table 3.

Moreover, when applied, these adjusted thresholds resulted in a more accurate classification of sarcopenia severity. The revised classification delineated probable sarcopenia, sarcopenia, and severe sarcopenia as per the EWGSOP2, and these were superior predictors of mortality risk when compared to the classifications according to EWGSOP1. Interestingly, the study discovered that LGS ≤ 0.8 m/s was a superior mortality risk predictor exclusively in individuals with probable sarcopenia.

In summary, the study accentuates the significance of LMS and LGS as potent predictors of mortality risk in older adults [49]. Furthermore, it suggests that the EWGSOP2 criteria might offer superior predictive accuracy for mortality risk compared to the earlier EWGSOP1 framework. These findings warrant a critical reevaluation of the existing diagnostic criteria and call for their refinement to enhance accuracy in mortality risk prediction.

## 4. Digital Health Technologies and IoT Devices for Sarcopenia Management

**Figure 1:**
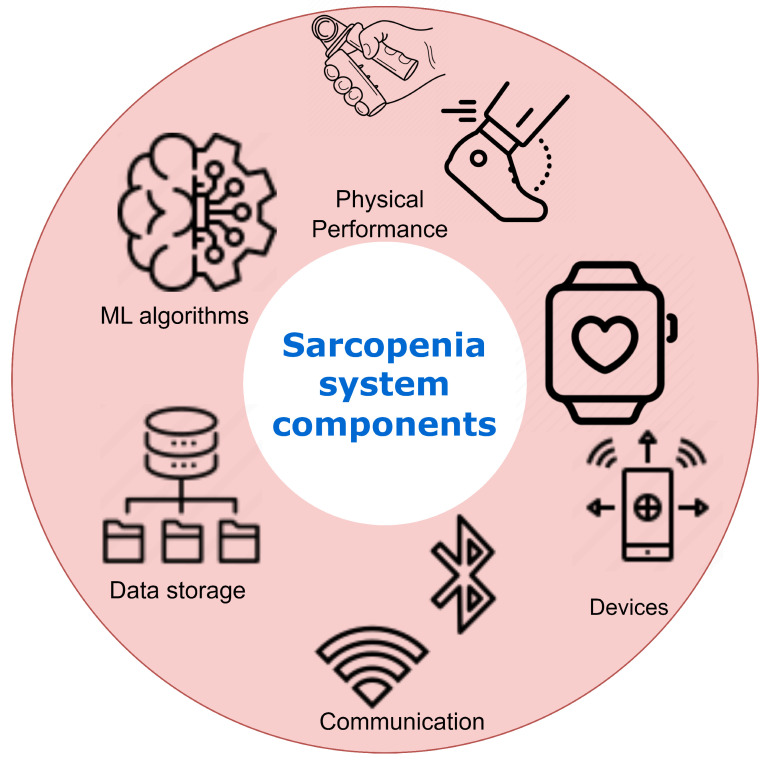
Sarcopenia system components. The sarcopenia monitoring system integrates wearable devices, edge computing, and AI to continuously collect multilayered health data, discern trends predictive of sarcopenia, and provide personalized recommendations to patients and clinicians.

Recent advancements in Information and Communication Technology (ICT) have introduced novel methodologies for assessing and analyzing sarcopenia. This survey article delineates a technical framework for monitoring and evaluating sarcopenia, utilizing cutting-edge ICT solutions. In the subsequent subsections, each constituent of the Sarcopenia ICT system is delineated, as illustrated in Figure 1.

### 4.1. Physical Activity Monitoring

This component involves using wearable devices like smartwatches and fitness trackers to continuously monitor physical activity levels, which are strongly associated with sarcopenia onset and progression. Key metrics tracked include step count, activity intensity, heart rate, etc. This allows longitudinal tracking of activity patterns predictive of sarcopenia.

### 4.2. IoT Integration

The system seamlessly integrates IoT-enabled devices like scales, blood pressure monitors, and pulse oximeters to regularly collect health parameters. Combined with activity data, these multilayered data provide insights into sarcopenia progression. The data are transmitted to a mobile app for aggregation and preprocessing.

### 4.3. Edge Computing Infrastructure

An edge computing infrastructure handles the continuous streams of monitored data. Edge computing allows real-time data processing to generate actionable insights and feedback for patients and clinicians. Machine learning models can discern trends predictive of sarcopenia.

### 4.4. AI and Machine Learning

AI and ML algorithms analyze heterogeneous datasets to recognize patterns in sarcopenia progression across patients. Patient-specific models can predict future changes and recommend interventions like exercise regimens and diet plans. The models improve continuously through retraining as more data are gathered.

### 4.5. User Interface and Clinical Feedback

A user-friendly interface provides patients and clinicians easy access to visualized data, detected trends, and AI-generated recommendations. Clinicians can validate insights and tweak intervention plans. Their feedback is used to retrain ML models.

### 4.6. Accelerometers

**Figure 2:**
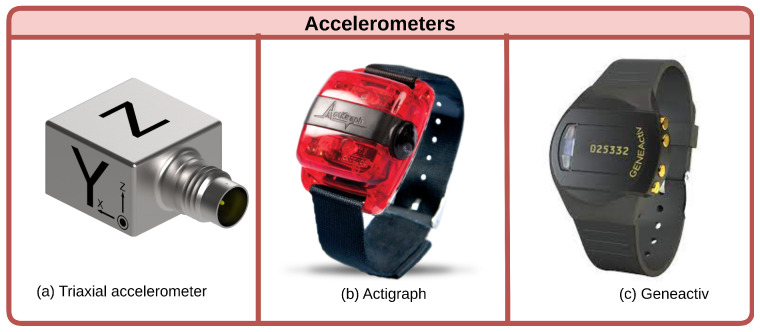
Accelerometer types. Triaxial accelerometers measure motion in three planes. Actigraphs specifically track metrics like step counts and activity intensities. Geneactivs provide raw acceleration data for sophisticated analysis.

Accelerometers offer an objective approach to measuring physical activity and sedentary behavior, providing valuable insights into sarcopenia, a condition characterized by reduced muscle mass and function that commonly afflicts the elderly. Multiple studies utilizing accelerometers have uncovered intriguing connections between activity levels and sarcopenia, although some discrepancies exist regarding diagnostic methods and dose–response relationships (Figure 2).

Several investigations have demonstrated reduced moderate-to-vigorous physical activity (MVPA) in sarcopenic versus non-sarcopenic groups [50,51,52,53,54]. However, the relationship between sedentary time and sarcopenia remains unclear. While Scott et al. found a decreased likelihood of low appendicular lean mass (ALM) with more sedentary time [50], Johansson et al. showed MVPA modulates this association [51]. Nevertheless, consensus is lacking on accelerometer cut-off points and sarcopenia definitions [51,55].

Dose–response analyses reveal intriguing but preliminary findings. Iwasaka et al. observed a plateau in the relationship between steps and sarcopenia risk below 10,000 steps [52]. Additional research on step count recommendations is warranted.

Longitudinal and intervention studies underscore the importance of physical activity in counteracting sarcopenia. Increased activity correlated with reduced adiposity, higher muscle mass and strength, and lower sarcopenia likelihood [56]. Accelerometer-based activity monitoring could aid sarcopenia prevention and management in elderly adults [54,55].

Overall, accelerometers enable the quantification of activity patterns relevant to sarcopenia. While diagnostic consensus and dose–response relationships require further elucidation, accelerometers have proven utility in epidemiological studies on sarcopenia’s complex etiology [57]. Standardizing methodology and accelerometer use will be key to developing effective interventions against this disabling condition.

### 4.7. Pedometers

Pedometers offer a practical means of objectively evaluating physical activity associated with sarcopenia, a condition of reduced muscle mass and function in the elderly. Multiple investigations demonstrate pedometer-measured step count as an insightful sarcopenia assessment and intervention metric [58].

Several studies reveal lower step counts in sarcopenic versus non-sarcopenic older adults. Using a validated accelerometer-based pedometer, Meier et al. found sarcopenic participants took fewer daily steps, spent more time sedentary, and showed worse physical function compared to active counterparts [59]. In a randomized controlled trial, Yuenyongchaiwat et al. implemented a pedometer-monitored walking regimen of 7500 steps per day to improve cardiopulmonary function in sarcopenic Thai adults successfully [58].

Additional research links reduced step count to heightened sarcopenia risk. In a year-long study, fewer steps and less moderate physical activity time correlated with lower muscle mass [60]. Yuenyongchaiwat and Akekawatchai demonstrated sarcopenia improvement in older adults instructed to walk ≥7500 steps daily [61].

While highlighting the utility of pedometers in sarcopenia assessment and activity monitoring, these studies establish associative rather than causal relationships. Further interventional research is required to delineate optimal step count recommendations and determine pedometer-based programs’ efficacy for sarcopenia prevention and treatment. Nevertheless, existing research suggests pedometers could prove valuable tools alongside accelerometers and other devices in elucidating and managing sarcopenia’s complex relationship with physical activity.

### 4.8. Inertial Measurement Units (IMUs)

Inertial measurement units (IMUs) offer a versatile option for evaluating physical functioning relevant to sarcopenia. These wearable sensors enable quantitative analysis of gait, balance, and mobility patterns by containing accelerometers, gyroscopes, and sometimes magnetometers. Multiple studies demonstrate IMUs’ utility in sarcopenia assessment and monitoring.

Several investigations utilize IMUs to distinguish sarcopenic from healthy controls through gait analysis. Ortega-Bastidas et al. demonstrated an IMU’s ability to assess fall risk during the Timed Up and Go test, highlighting the technology’s portability and cost-effectiveness [62]. Employing various classification algorithms on IMU data, Kim et al. identified sarcopenic patients with 95% accuracy based on locomotion features [63]. In another study, Kim et al. uncovered associations between IMU-derived gait variables like stride length and gait speed with muscle mass, strength, and function [64].

Beyond gait analysis, IMUs enable the quantification of other relevant functional parameters. Kim et al. used IMUs to characterize diminished endurance, mobility impairments, and increased dependence in sarcopenic adults during daily living [65]. Byun et al. developed an IMU algorithm to estimate walking speed in the elderly with high accuracy, demonstrating IMUs’ potential for long-term monitoring [66].

These studies provide consistent evidence for IMUs’ versatility in assessing muscle deterioration and functional limitations related to sarcopenia. While additional research is needed to optimize algorithms and standardize protocols, IMUs show promise as portable, cost-effective tools that complement existing sarcopenia evaluation and monitoring methods. Their capacity to measure gait, balance, endurance, and mobility deficits in real-world settings could prove invaluable in elucidating sarcopenia’s impact on elderly patients’ physical performance and independence.

### 4.9. Pressure-Sensitive Insoles

Instrumented insoles show considerable potential for assessing gait, balance, and fall risk in older adults. Several studies have explored pressure-sensitive insoles to analyze the center of pressure and plantar pressures during locomotion. Kraus et al. [67] and Anzai et al. [68] demonstrated that data from instrumented insoles can effectively identify physical frailty and sarcopenia risk through gait analysis. Compared to traditional assessments like SARC-F and Timed-Up-and-Go, machine learning models using insole data showed improved detection of frailty status.

Beyond frailty detection, pressure-sensitive insoles also enable an analysis of biomechanical factors that may predispose individuals to injury during physical activity. For instance, Draganich et al. [69] used insoles to examine changes in the center of pressure during the transition to minimalist running shoes as a way to identify runners at risk of stress fractures. Atreya et al. [70] assessed how kitchen task height impacted plantar pressures in older adults, suggesting the potential for using insoles to monitor age-related physical performance declines.

Several research groups have developed and tested insole-based sensors using different technological approaches. Martini et al. [71] created an optical plantar pressure sensor prototype for integration with wearable robotics. Silva et al. [72] built an instrumented insole with 10 pressure sensors for gait and balance analysis. Subramaniam et al. [73] reviewed various systems and their efficacy in assessing fall risk. In addition to sensing capabilities, the physical design of instrumented insoles may also contribute to fall prevention through improved balance and stability.

Pressure-sensitive insoles enable quantitative assessment of older adults’ gait, balance, and fall risk. Continued technological development and research on analytic techniques point to the growing potential for instrumented insoles in the early detection of age-related physical performance decline and timely intervention to improve outcomes. Further validation of insole systems against gold-standard tools and prospective studies are still needed to translate this emerging technology into clinical practice.

### 4.10. Smartphone Applications

Smartphone applications (SAs) are emerging as innovative tools for sarcopenia management by providing nutritional support, fall prevention, social engagement, and exercise programs [74,75]. Smart appliances like refrigerators that monitor food contents could potentially enhance nutritional status, while mobile apps for fall detection and prevention address the high fall risk in sarcopenic populations [74]. Applications also mitigate social isolation associated with sarcopenia progression [74]. However, most nutrition apps are not validated and emphasize restriction over optimal protein and nutrient intakes for muscle health [75]. Further high-quality research is needed on virtual reality, wearables, robotics, and neuromuscular electrical stimulation to boost capacity for daily living activities and physical activity in sarcopenic adults [75].

Emerging work has focused on validating mobile apps for sarcopenia screening and detection. Montemurro et al. [76] validated an iPhone app measuring sit-to-stand power through video analysis plus muscle mass. Early detection of pre-sarcopenia enables timely interventions to prevent functional decline and adverse outcomes [76]. This hand-held assessment of muscle power is as accurate as laboratory methods and allows rapid community-based screening [76]. Remote sensing devices like BandPass provide personalized feedback on resistance exercise compliance and progress by capturing quantitative muscle strength data [77]. HTSMayor software estimates appendicular muscle mass using anthropometry or DXA and demonstrates high diagnostic accuracy for sarcopenia versus DXA [78].

SAs represent promising tools for sarcopenia management. While early research has focused on screening and detection, further development and validation are needed for nutritional, social, fall prevention, and exercise apps tailored to sarcopenic populations. Wearables and remote monitoring devices also provide personalized feedback and progress-tracking opportunities. Overall, mobile health innovations may increase accessibility and optimize sarcopenia interventions.

### 4.11. Challenges and Future Work

Accelerometers provide objective measurements of physical activity levels and sedentary behavior valuable for diagnosing and monitoring sarcopenia [55]. However, the specific variables associated with sarcopenia can depend on the assessment tools and criteria used. Pedometers are limited by their indirect measurement of physical activity and muscle strength. However, combining pedometer data with other metrics like muscle quality could aid sarcopenia research [79].

Inertial measurement units (IMUs) can quantify mobility but are limited in their measurement of muscle strength and power. Improving IMU accuracy and affordability could enhance their utility for detecting changes in the range of motion and muscle function. Pressure-sensitive insoles provide insight into balance and gait but are limited in their direct assessment of muscle strength and power. Combining insoles with other tools like electromyography may allow a more comprehensive evaluation of muscle function [80].

Smartphone applications provide easy tracking of physical activity but rely on self-reporting and indirect strength measures. Integrating smartphone apps with wearable sensors or remote monitoring could improve measurement accuracy [81]. Overall, technology-based tools show significant potential for assessing sarcopenia but require ongoing research to refine accuracy, reliability, accessibility, and integration with other assessment techniques. Standardization of protocols and validation in diverse populations is also needed to fully realize their promise in sarcopenia diagnosis and management.

## 5. Healthcare Platform

### 5.1. Sarcopenia Datasets

Several studies have focused on identifying important variables associated with sarcopenia using datasets from specific populations. Zhang et al. [82] analyzed data from over 4000 older Chinese adults and found that a Wide and Deep neural network model incorporating 12 routine clinical variables (e.g., age, arm circumference, liver enzymes) could effectively predict sarcopenia status. Castillo-González et al. [83] followed 166 Mexican seniors over 6 months and applied machine learning to conclude that age, blood pressure, nutrition, comorbidities, and sodium levels were most indicative of sarcopenia level and progression. In an Italian cohort [84], albumin, C-reactive protein, vitamin D, and folate emerged as key biomarkers associated with sarcopenia, muscle mass, and strength, see Table 4 below.

Other studies have specifically examined whether analysis of pulse waveforms and spectral features using signal processing and machine learning methods could provide a simple approach to screen for possible sarcopenia. Wu et al. [85] found several differences in harmonic content and variability between 133 sarcopenic and robust seniors, and their models could discriminate based on 1-minute radial pulse measurements.

There has also been interest in developing prognostic models to identify those at risk of sarcopenia for early intervention. Cernea et al. [86] constructed a model for 200 older adults incorporating gait speed, BMI, fat-free mass, oxidative stress, depression, and medication use that could effectively predict sarcopenia status. Their results highlight the multifactorial nature of sarcopenia encompassing muscle mass, strength, function, and systemic factors.

Nationwide surveys of community-dwelling older adults in Korea have provided population-level insights, with sarcopenia prevalence estimates ranging from 6.6% to 13.3% using different diagnostic criteria [87,88]. These large datasets have elucidated shifting patterns in sarcopenia screening when using different muscle mass and physical performance measures [87]. Research has also leveraged datasets from specific clinical populations, including bariatric surgery patients, to elucidate sarcopenia risks and the impacts of interventions [36].

Beyond foundational prevalence data, datasets integrating detailed patient information have enabled predictive modeling of sarcopenia risk using machine learning. Models based on body composition, blood biomarkers, and dietary intake data can identify significant sarcopenia risk factors like low BMI, elevated BUN, and insufficient protein intake [89].

Longitudinal datasets have also provided insights into sarcopenia progression over time. Repeated measurements across treatment timelines for bariatric surgery patients reveal granular impacts of surgical interventions and exercise on body composition and strength [36]. Population-based surveys capturing health-related quality-of-life measures have characterized sarcopenia’s effects on critical patient-centered outcomes [88].

Sarcopenia research has been strengthened by diverse datasets quantifying muscle mass, strength, physical function, patient-reported outcomes, and predictive risk factors in both cross-sectional and longitudinal formats [36,87,88,89]. These datasets underscore the multifaceted nature of sarcopenia and the need for regular, comprehensive assessments to elucidate prevalence, predictors, disease progression, and intervention efficacy across populations. Continued accrual of rich, heterogeneous datasets will further enhance sarcopenia characterization and support personalized risk stratification and care.

### 5.2. Cloud Computing in Healthcare

In recent years, cloud computing has emerged as a pivotal technology for enabling remote healthcare services and improving patient care. Several studies have investigated techniques for developing robust and secure cloud-based systems tailored to healthcare. Zhang et al. [90] proposed an isolation mechanism to ensure performance isolation between tenants in a multitenant IoT cloud platform for smart healthcare. Their scheduling algorithm provided effective feedback control of asynchronous data flows to achieve tenant isolation, reducing message delays. While this mechanism could enhance service quality for patients, future work on preserving privacy with isolation was noted.

Complementarily, Dang et al. [91] provided a comprehensive survey of the IoT framework for healthcare, reviewing the topology, structure, platform components, and key concepts, including fog computing, ambient assisted living, wearables, and blockchain. Trends and applications across patient monitoring, clinical operations, and fitness were summarized alongside security considerations and policies promoting IoT healthcare. However, issues like system development, resource management, and security/privacy remained.

Critically examining the security dimension, Masud et al. [92] proposed a robust and lightweight access scheme for cloud-based E-healthcare services to curb threats and prevent unauthorized access. Their scheme enabled end-to-end encryption and access control based on stakeholder identity to protect sensitive patient data in the cloud. While simplicity and robustness were strengths, further analysis of encryption mechanisms could be valuable. To boost the availability of quality healthcare services globally, Sahu et al. [93] designed an innovative remote patient monitoring system (RPMS) leveraging IoT for real-time health monitoring, abnormality detection, and alert notification. This system was positioned to potentially overcome challenges in delivering comprehensive healthcare for remote and underserved populations. Additional investigation into deploying such systems was recommended.

Providing a holistic perspective, Mohemmed Sha et al. [94] detailed how cloud computing has transformed healthcare data management, outlining a framework to harness relevant services from major providers to manage data flows. As a reference model, an architecture using Amazon cloud services was presented. While informative, further research into optimizing configurations for healthcare-specific needs could be useful.

Cloud computing presents a novel approach for enhancing remote healthcare delivery for sarcopenia through recent advances in multitenant platforms, comprehensive architectures, secure access schemes, real-time monitoring, and tailored data management.

### 5.3. Blockchain in Healthcare

Blockchain technology has emerged as a transformative solution to address longstanding challenges in healthcare information systems. As [95] discuss, blockchain implementation could enhance secure electronic medical record management, drug supply chain traceability, and interoperability across institutions. They propose a blockchain-based hospital system prototype where each patient’s data reside in an interconnected block, enabling interaction with blockchain applications for decentralized data sharing.

Other studies have explored blockchain-based healthcare management systems leveraging smart contracts and decentralization for efficient, secure data handling. Research in [96] introduced a hybrid permission–permissionless blockchain model called Med-PPPHIS to manage personal health information through on-chain medical data tokens. However, they acknowledge limitations around inefficient chain queries, revocation of access permissions, and lack of dynamic public chain selection.

To facilitate ubiquitous-to-electronic health record conversion, in the study of Chelladurai and Pandian, Ref. [97] designed a blockchain system using smart contracts to regulate access to fragmented records across providers. It offers a distributed ledger for patients, guaranteeing security through cryptographic hashing while enabling seamless physician access. System performance was validated through metrics like resource use and transaction latency.

Similarly, Ref. [98] developed a multi-layered blockchain system for managing personal health information. It comprises blockchain, IoT, application, and adapter layers to address security, privacy, and interoperability challenges.

Blockchain solutions are transforming healthcare information systems by enhancing security, interoperability, and data sharing. While some limitations around scalability and flexibility exist, ongoing research aims to optimize blockchain-based approaches for managing electronic medical records. This technology promises to improve healthcare delivery by securing information exchange between patients and providers.

### 5.4. Edge in Healthcare

Edge computing is emerging as a promising technology for enhancing healthcare services through improved data collection, processing, and analytics. Recent studies have proposed innovative frameworks leveraging edge computing to enable more personalized, efficient, and secure healthcare systems.

For instance, Xie et al., (2022) presented a model that collects and stores digital healthcare data using a combination of soft computing and edge-driven multimodal systems. The model acts as a catalyst for personalizing healthcare by providing a comprehensive patient data perspective. Soft computing techniques address the complexities of healthcare data and improve the efficiency and effectiveness of services. The integrated file management system enables a feedback loop for deeper patient data insights.

Similarly, Jazaeri et al., (2023) introduced an edge computing approach to boost IoT healthcare quality, combining caching and classification techniques. Built on software-defined networking, it aims to provide timely, accurate information by improving efficiency and minimizing latency. A multi-criteria caching algorithm considers vital factors like data size, frequency, and significance. Spectral clustering groups patients based on medical records. These optimizations enhance performance, reduce latency, improve quality of experience, and lower costs.

Singh et al., (2023) designed an Edge of Things framework for secure, efficient health monitoring using edge computing. The tripartite architecture comprises edge, fog, and cloud layers performing data collection, processing, storage, and analysis. Security measures like encryption, authentication, and access control safeguard privacy. Key features include real-time monitoring, secure access, clustering, encryption, reduced overhead, and the three-layer model.

Liu et al., (2022) proposed integrating multimedia, multimodal sensing, and edge computing to optimize data in personalized healthcare supply chains. Multimedia and multimodal sensing gather diverse data, while edge computing enables real-time processing to improve supply chain efficiency. Benefits include interface optimization, efficient analysis, disease prevention, diagnosis, personalized care, social sensing, and ambient assisted living.

Edge computing shows great promise for advancing healthcare services through localized, real-time data processing. Key applications include personalization, security, efficiency, latency reduction, and improved analytics. Hybrid systems blending edge computing with IoT, soft computing, and multimodal data offer robust healthcare solutions. Further research can explore the optimization of these architectures.

## 6. Machine Learning Algorithms

This section will consider some aspects of machine learning algorithms in detecting sarcopenia. Logistic regression, a critical statistical technique, is used to predict the likelihood of sarcopenia by incorporating multiple predictor variables. While models like the ones proposed by Kaur et al., Agnes et al., and Yin et al. show their potential, the balance between statistical precision and practical implementation needs careful consideration. Moving on to machine learning algorithms, the Support Vector Machine (SVM) algorithm has demonstrated promise in sarcopenia prediction. However, it faces some limitations, like data dependency, feature selection sensitivity, and generalizability and interpretability issues. The Random Forest (RF) algorithm shows potential in managing extensive datasets and non-linear relationships, despite challenges with smaller datasets and overfitting. Gradient Boosting Machines (GBM) and K-nearest neighbors (KNN) have also been used for sarcopenia prediction, offering promising results. Despite the pros and cons of each method, these models provide crucial insights into sarcopenia risk prediction, significantly improving healthcare for older adults.

**Figure 3:**
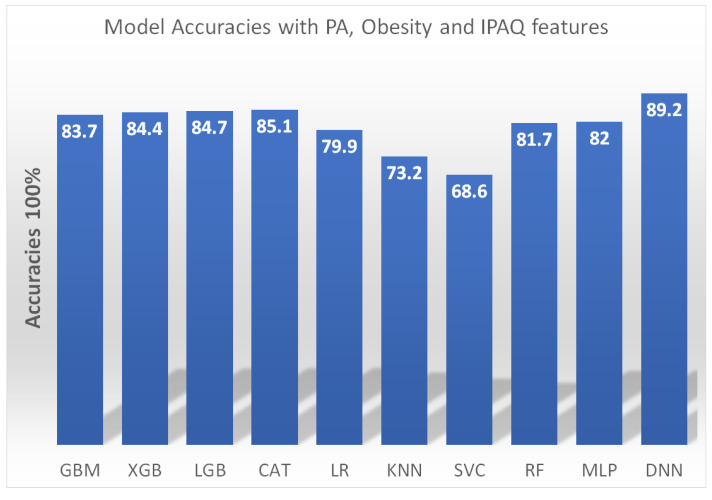
Accuracy of machine learning models for sarcopenia detection using physical activity, obesity, and IPAQ features. A deep neural network (DNN) model achieved the highest accuracy of 89.2%. Gradient boosting techniques (GBM, XGB, LGB, CAT) and random forest (RF) also performed well with accuracy above 81%. A multilayer perceptron (MLP) model scored 82% accuracy. Logistic regression (LR) and k-nearest neighbors (KNN) were weaker baseline models. The results demonstrate the feasibility of sarcopenia detection via machine learning applied to multidimensional health data [99].

Furthermore, as illustrated in Figure 3, a comparative analysis was conducted on various machine learning-based predictive models for the detection of sarcopenia, specifically utilizing physical activity (PA), obesity measures, and the International Physical Activity Questionnaire (IPAQ) as the primary predictor variable [99]. Model performance was compared based on accuracy in predicting sarcopenia status. The gradient boosting machine (GBM), extreme gradient boosting (XGB), light gradient boosting machine (LGB), catboost (CAT), logistic regression (LR), k-nearest neighbors (KNN), support vector classifier (SVC), random forest (RF), multilayer perceptron (MLP), and deep neural network (DNN) models were trained and tested.

The DNN model achieved the highest accuracy of 89.2%, demonstrating the capability of deep learning methods for sarcopenia detection with multidimensional health data. The ensemble models GBM, XGB, LGB, and CAT performed well with accuracy scores above 83%, showing the utility of boosting techniques. The baseline LR model had an accuracy of 79.9%. In comparison, the KNN model performed relatively poorly with an accuracy of just 73.2%. Overall, the results highlight the feasibility of applying advanced machine learning approaches to leverage data from wearables, questionnaires, and health assessments for automated sarcopenia screening and diagnosis. Further research can build on these findings to develop more sophisticated and generalizable predictive models.

### 6.1. Logistic Regression

Logistic regression has emerged as a powerful statistical technique for predicting sarcopenia risk and onset amongst aging populations. By incorporating multiple predictors like age, sex, body composition, and physical activity, logistic models can estimate the individual likelihood of developing sarcopenia [100,101,102]. These predictive models enable more targeted screening and early interventions.

A notable study by Kaur et al. [100] demonstrates the potential of logistic regression in evaluating sarcopenia and frailty. While specific variables were not defined, their model achieved a remarkable 97.69% accuracy in predicting outcomes. However, such high precision warrants further analysis of real-world applicability. In contrast, Agnes et al. [101] developed a multi-logistic model incorporating key predictors like BMI and calf circumference to predict sarcopenia. With 80% sensitivity and 70% specificity, this model shows promise as a practical screening tool. Expanding on this, Yin et al. [102] combined logistic regression and nomogram visualization to accurately predict individual sarcopenia risk, highlighting clinical utility.

Challenges in logistic regression modeling for sarcopenia may include issues related to data quality, sample size, and variable selection. For example, the use of computed tomography images to assess skeletal muscle index may be subject to measurement error. Additionally, small sample sizes may limit the ability to detect significant associations between sarcopenia and performance status. Finally, selecting the most relevant independent variables to include in the model can be challenging, as there may be many potential predictors of poor performance status in sarcopenia [103].

While forms and applications vary, logistic regression models consistently provide crucial insights and value in understanding, predicting, and combating sarcopenia. The capacity to distill risk factors and convert statistical findings into practical tools underscores the power of logistic regression in sarcopenia research and care. Synthesis of these studies suggests logistic modeling will continue to advance early detection and intervention efforts.

### 6.2. Support Vector Machine

The support vector machine (SVM) algorithm has demonstrated utility within sarcopenia prediction as an effective machine learning approach for classification and modeling. Studies leveraging SVM have generated predictive models with notable accuracy. For instance, Ko et al. [104] combined SVM analysis of data from inertial measurement units during walking to achieve 95% prediction accuracy. This highlights the potential of SVM-based kinematic analysis. Similarly, Kim [105] formulated predictive models for sarcopenia using public health data and multiple algorithms, including SVM. While the LightGBM algorithm performed best in this study with 85.2% accuracy, the SVM model also surpassed 80% accuracy. This comparison of SVM and LightGBM illuminates the need for further research into optimal algorithms. Nonetheless, SVM has exhibited considerable promise, as underscored by Seok and Kim [99].

Expanding the discussion towards the limitations of using SVM for sarcopenia prediction, certain impediments become evident:

- As underlined by Seok and Kim [99], one primary constraint lies in the data limitation. SVM is inherently dependent on ample datasets for effective model training. However, accumulating extensive data for sarcopenia prediction is daunting, as it necessitates specialized apparatus and expertise.
- Kang et al. [89] elaborate on the sensitivity of SVM towards feature selection. The careful selection of appropriate features becomes a pivotal aspect of achieving optimum accuracy in sarcopenia prediction.
- E Kim [105] raises a valid concern about the generalizability of SVM models. Due to the specific datasets used for model training, SVM models may lack the ability to generalize across new datasets or varied populations. This could potentially limit the model’s capacity to encapsulate the complete variation of sarcopenia across disparate populations.
- A significant interpretability issue is raised by Castillo et al. [106]. They highlight that SVM models are frequently seen as “black box” models, which pose a challenge in interpreting the path leading to the model’s predictions. This could hinder our understanding of the underpinning mechanisms of sarcopenia and the development of targeted interventions.

Although limitations remain, SVM has emerged as a valuable tool within multifaceted prediction frameworks for sarcopenia. The algorithm’s classification prowess enables impactful modeling, particularly when combined collaboratively with other machine learning techniques. Ongoing optimizations to SVM and model integration will likely advance sarcopenia screening and care. While challenges persist, SVM’s utility in this domain should not be discounted, and continued research is warranted.

### 6.3. Random Forest

The random forest (RF) algorithm has demonstrated increasing utility within sarcopenia prediction research. RF uses many decision trees, each trained on bootstrap samples with stochastic elements, to enable robust generalization [99]. This machine learning approach shows promise for sarcopenia modeling.

Specific applications include Kang et al. [89], who compared RF to other classifiers using 17 risk factors. Their 2000 tree RF model identified important predictors, though logistic regression performed best overall. Beyond this, Yoon et al. [107] applied RF to predict sarcopenia in cancer patients, while Seok and Kim [99] used physical activity data and RF to estimate sarcopenia likelihood in the elderly.

Generally, RF excels at managing extensive datasets and nonlinear relationships, making it well-suited for integrating diverse clinical and anthropometric sarcopenia predictors [99]. However, limitations exist, including poorer performance on small datasets, difficulty handling missing data, and overfitting risks. Cautious refinement is needed, but RF remains a powerful option for sarcopenia modeling.

Ongoing research should explore optimal RF configurations, predictor selection, missing data techniques, and model tuning to maximize predictive performance while minimizing overfitting. Though challenges remain, RF provides a flexible framework to leverage multiple data types for sarcopenia screening and risk stratification. Synthesis of existing research highlights the promising role of RF-based modeling in this domain.

### 6.4. Gradient Boosting Machines

Gradient boosting machines (GBMs) have emerged as versatile machine learning techniques for sarcopenia prediction. GBM uses an ensemble approach, integrating numerous weak decision tree models into a robust classifier [99]. The algorithm’s unique boosting methodology has shown utility for sarcopenia modeling.

By repeatedly training models on diverse activity data and other attributes, GBM introduces randomness while optimizing based on error metrics [89,99,108]. Comparatively, GBM achieves prediction accuracy on par with logistic regression and random forest, with an AUC of 0.78–0.85 [99]. However, further validation of GBM sarcopenia models is needed.

Various informative features have been incorporated, including physical activity variables like gait speed, strength, and muscle mass [99], nutritional factors such as BMI and protein intake [108], and demographics like age and sex [89]. CT-derived radio mic muscle features also show promise [108]. These multifaceted data aid sarcopenia risk identification.

Specifically, activity-related parameters like BMI, walking speed, strength, muscle mass [99], and CT radiomic features [108] have been integrated into GBM models. Such data play a key role in developing predictive sarcopenia frameworks to identify risks in aging populations.

GBM is a versatile approach leveraging ensemble decision tree modeling for sarcopenia prediction. Despite needing further validation, GBM demonstrates comparable performance to other machine learning techniques. Ongoing research should refine feature selection and model optimization to maximize GBM predictive utility in sarcopenia screening.

### 6.5. K-Nearest Neighbors

The K-nearest neighbors (KNN) algorithm has emerged as a key machine learning approach for sarcopenia prediction. As a non-parametric method, KNN categorizes new data points based on the classification of their closest k neighbors in the training data [99,109]. This enables KNN to distinguish sarcopenic versus non-sarcopenic cases using activity and other predictor variables.

Moreover, KNN is often integrated into ensemble frameworks along with logistic regression, support vector machines, random forest, and gradient boosting machines to develop robust sarcopenia prediction models [89,99,108]. The synergistic use of KNN underscores its utility beyond a standalone tool.

Comparatively, KNN demonstrates prediction accuracy on par with leading machine learning algorithms for sarcopenia screening [89,99]. However, some methods like XGB have shown slightly better performance in select studies [89,110]. This highlights the nuanced nature of algorithm selection for optimal outcomes.

KNN is a versatile machine learning approach for sarcopenia prediction, especially when combined with other classifiers. Ongoing research should refine parameter selection and ensemble integration to maximize KNN’s utility. Though performance varies across applications, KNN remains a valuable option for developing predictive sarcopenia frameworks.

### 6.6. Explainable AI

Explainable AI techniques are critical for building trustworthy AI systems, especially in sensitive domains like healthcare. For example, when developing algorithms to detect sarcopenia, a muscle loss condition in older adults, explainability enables physicians to understand and validate the algorithm’s reasoning. This increases adoption, as doctors can trust the technology. Explainable sarcopenia detection systems could extract interpretable features from medical images and clinical data. They could also provide examples of similar cases and visual highlighting of affected regions. Overall, explainable AI provides transparency into how algorithms make decisions. This allows clinicians to feel confident leveraging AI for enhanced sarcopenia screening and treatment [111].

## 7. Research Challenges

Sarcopenia, a prevalent condition among the elderly, is typified by the age-related degradation of muscle mass and strength. It is a multifaceted condition arising from an intricate interplay of genetic, lifestyle, and environmental factors. Sarcopenia may precipitate numerous adverse health consequences, encompassing an elevated risk of falls, disability, and mortality. Therefore, the demand for efficacious strategies for managing and preventing sarcopenia is undeniable. Wearable technology, such as fitness trackers and smartwatches, offers the potential for invaluable insights into the progression of sarcopenia and may aid individuals in observing and controlling their condition. However, the use of wearable technology for sarcopenia research presents several challenges.

### 7.1. Machine Learning Challenges in Sarcopenia Detection

While machine learning techniques show promise for improving sarcopenia definition and diagnosis [2], several key challenges remain:

*Data Accessibility:* Limited data accessibility persists, as datasets are often small-scale and may contain incomplete entries due to inconsistent body measurement collection [2]. This can restrict model efficiency and generalizability.

*Data Imbalance:* Class imbalance is common, such as higher sarcopenia prevalence in females [2]. Although techniques like the Synthetic Minority Oversampling Technique (SMOTE) can balance classes, this risks over-generalization.

*Feature Selection:* Optimal feature selection is unclear, as excessive features reduce clinical utility while too few lower accuracy [2]. Moreover, feature combinations impact classifier performance differently, complicating comparisons.

Sarcopenia machine learning faces hurdles, including scarce quality data, class imbalance, and ambiguous feature selection. Moving forward, larger datasets, synthetic balancing methods, and feature optimization will be vital to maximizing model accuracy while retaining clinical applicability. Though challenges exist, machine learning presents exciting opportunities to advance sarcopenia assessment.

### 7.2. Challenges in Physical Performance Measurement

Several key challenges exist in the physical performance measurement of sarcopenia:

*Absence of Consensus or Standardization:* The absence of consensus on sarcopenia definition and diagnostic criteria hinders result comparison between studies, as groups like EWGSOP and AWGSOP use distinct measures [112,113].

*Restricted Access to Reliable and Validated Diagnostic Equipment:* Limited access to validated diagnostic equipment, especially in primary care, impedes early risk identification [114].

*Measurement of Physical Activity, Sedentary Time, and Fitness:* Accurately measuring physical activity, sedentary time, fitness, and strength is difficult. Self-reported physical activity can involve bias, while performance tests may not be universally feasible [59].

*Patient Response to Potential Therapies:* Evaluating diagnostic tools’ ability to predict patient therapeutic response is challenging [113].

Key obstacles around standardization, imaging techniques, equipment availability, lifestyle factor measurement, and therapy prediction must be addressed to enhance sarcopenia assessment. Consensus-building and access expansion will be instrumental in overcoming these physical performance measurement challenges.

## 8. Conclusions

In conclusion, this survey provides a comprehensive overview of sarcopenia research, focusing on detection and management approaches leveraging modern technologies. However, several challenges remain in applying consensus definitions, standardized methodologies, and optimal technologies.

A key challenge is the lack of universal criteria for defining and diagnosing sarcopenia, as evidenced by distinct cut-off values and assessment protocols from EWGSOP and AWGSOP. This impedes comparative analyses and knowledge synthesis across studies. Standardizing sarcopenia assessment through collaborative initiatives could enable more robust insights.

Additionally, while machine learning shows promise for automating sarcopenia evaluation, limitations around data availability, labeling, and feature optimization must be addressed. Wearable sensors may help collect informative real-world mobility data to track sarcopenia progression and guide interventions. However, analytical techniques need advancement to glean clinically valuable insights from these data.

Emerging technologies like blockchain and edge computing may enhance privacy and decentralization for health data analytics. However, scalability and latency issues remain for processing high-volume medical datasets. More dynamic, optimized systems are needed to fully realize the potential of these technologies in sarcopenia management.

Future investigations into computational methods for sarcopenia diagnosis show promise. The application of fuzzy logic approaches may improve the accuracy of sarcopenia detection by accounting for the inherent imprecision in defining this syndrome. The fuzzy set theory provides mathematical formalism to handle such linguistic uncertainty [115]. Additionally, deep learning techniques like convolutional neural networks (CNNs) offer powerful pattern recognition capabilities that are well-suited for medical imaging applications. CNNs and other deep networks have achieved state-of-the-art performance in diverse computer vision tasks. Leveraging these advanced artificial intelligence methods could enable robust, automatic sarcopenia quantification and staging from routinely acquired CT, MRI, or ultrasound scans. This would facilitate large-scale screening, enhance reliability compared to manual evaluation, and reduce costs [116,117,118,119,120]. Further research should explore fuzzy logic and deep learning for sarcopenia diagnosis and validate these techniques on diverse, clinically relevant datasets.

This survey underscores key innovations and persisting challenges in sarcopenia detection and care. Opportunities exist for accelerating research through data sharing, advancing analytics, and strategically implementing technologies balanced with clinical needs. Additional coordinated efforts to standardize methodologies, collect representative data, and translate technical innovations into patient care will help address current gaps and drive future sarcopenia management.

## Figures and Tables

**Table 1:** Literature review methodology.

Review Methodology	Details
Literature Search	Google Scholar Web of Science ScienceDirect
Inclusion Criteria	Peer-reviewed papers Published in the last 3 years English language Original research and review articles
Information Extraction	Key data extracted into a standardized form Results collated and compared
Synthesis Approach	Grouped by themes Chronological analysis of research trends

**Table 2:** Cut-offs as per the Asian Working Group for Sarcopenia.

Criteria	Measurements
Low Muscle Mass	DXA: <7.0 kg/m${}^{2}$ in men and <5.4 kg/m${}^{2}$ in women
	BIA: <7.0 kg/m${}^{2}$ in men and <5.7 kg/m${}^{2}$ in women
	DXA (NIH criteria): <0.789 kg/BMI for men and <0.512 kg/BMI for women
Low Muscle Strength	Grip Strength: <28 kg in men and <18 kg in women
Low Physical Performance	Gait Speed: ≤0.8 m/s

**Table 3:** Cut-offs as per the European Working Group for Sarcopenia.

Criteria	Measurements
LMM (Low Muscle Mass)	ASM (Appendicular Skeletal Muscle mass) divided by height squared: <7.0 kg/m${}^{2}$ for men and <5.7 kg/m${}^{2}$ for women
LMS (Low Muscle Strength)	Grip Strength: <27 kg for men and <16 kg for women
Low Physical Performance	Gait Speed: <0.8 m/s

**Table 4:** Sarcopenia datasets.

Study	Dataset Description	Population	Measurements	Key Findings
Zhang et al. [82]	West China Health and Aging Trend (WCHAT)	4057 people aged ≥ 50	Age, arm circumference, liver enzymes	12 routine clinical variables could effectively predict sarcopenia status
Castillo-Gonzalez et al. [83]	Baja California, Mexico	166 patients, mean age 77.24	Medical history, tests, comorbidities, functional capacity, nutrition status, biochemical data, and sociodemographics	The Decision Tree classifier on 5 key variables achieved high accuracy in assessing sarcopenia status and severity
Zupo et al. [84]	Clinical records and fluid markers, northern (Pavia) and southern (Apulia) Italy	1971 adults aged > 65	Low muscle mass, low muscle strength, and/or low physical performance according to EWGSOP2 guidelines.	Key variables identified include muscle mass, strength, sex, inflammation, and nutritional biomarkers
Wu et al. [85]	Tri-Service General Hospital (TSGHIRB 2-108-05-161)	133 subjects aged 71 years on average	Dynapenia, presarcopenia, and sarcopenia groups based on muscle strength, gait speed, and muscle mass measurements	Effectively discriminate possible sarcopenia subjects based on the noninvasive pulse measurements
Carnea et al. [86]	FRADEA (Frailty and Dependence in Albacete)	200 people aged ≥ 70	Fat-free mass, gait speed, body mass index, oxidative stress biomarkers, presence of depression, and use of proton pump inhibitors	The multifactorial nature of sarcopenia, with muscle mass, function, and quality as well as systemic factors like inflammation and oxidative stress contributing
Kim et al. [87]	Nationwide Korean Frailty and Aging Cohort Study (KFACS)	2123 community-dwelling older adults aged 70–84	Handgrip strength, usual gait speed, appendicular skeletal muscle mass	Higher prevalence of sarcopenia under AWGS 2019 definitions compared to AWGS 2014
Santos et al. [36]	Dataset on skeletal muscle mass index, body composition and strength in bariatric patients	46 bariatric surgery patients aged 18–60	Skeletal muscle mass index, body composition, strength	Provided a granular view of the impact of bariatric surgery and exercise on sarcopenia risk
Sun et al. [88]	2008 to 2011 Korean National Health and Nutrition Examination Survey	4937 adults aged 60 and older	Dual-energy X-ray absorptiometry scan	Prevalence of sarcopenia at 6.6%, with men more likely than women
Kang et al. [89]	Medical records of Korean postmenopausal women	4020 postmenopausal women	BMI, BUN, RBC count, dietary factors (water, fibre, protein intake)	Identified significant risk factors for sarcopenia using machine learning algorithms

## Abbreviations

The following abbreviations are used in this manuscript:

**Abbreviation**	**Explanation**
EWGSOP	European Working Group on Sarcopenia in Older People
AWGSOP	Asian Working Group for Sarcopenia
DEXA	Dual-energy X-ray absorptiometry
CT	Computed tomography
MRI	Magnetic resonance imaging
ADMA	Asymmetric dimethylarginine
SPPB	Short Physical Performance Battery
TUG	Timed up and go test
5TSTS	Five times sit-to-stand test
MVPA	Moderate-to-vigorous physical activity
ALM	Appendicular lean mass
SMOTE	Synthetic Minority Oversampling Technique
SVM	Support vector machine
RF	Random forest
GBM	Gradient boosting machines
KNN	K-nearest neighbors
IMU	Inertial measurement unit
IoT	Internet of Things
ICT	Information and communication technology
AI	Artificial intelligence
ML	Machine learning
